# 4q22.1 Contributes to Bone Mineral Density and Osteoporosis Susceptibility in Postmenopausal Women of Chinese Han Population

**DOI:** 10.1371/journal.pone.0080165

**Published:** 2013-11-21

**Authors:** Haojie Yang, Bo Zhang, Jialin Zhu, Dan Liu, Fanglin Guan, Xijing He

**Affiliations:** 1 The School of Life Science and Technology, Xi'an Jiaotong University, Xi'an, China; 2 The Second Department of Orthopedics, the Second Affiliated Hospital, College of Medicine, Xi'an Jiaotong University, Xi'an, China; 3 The Fifth Hospital of Xi'an, China; 4 Key Laboratory of Environment and Genes Related to Diseases, Ministry of Education, Xi'an, China; 5 Institute of Human Genomics & Forensic Sciences, Xi'an, China; Kunming Institute of Zoology, Chinese Academy of Sciences, China

## Abstract

Osteoporosis is a multifactorial disease in which genetic determinants are modulated by hormonal, environmental and nutritional factors. An important clinical risk factor in the pathogenesis of osteoporosis is the presence of genetics polymorphism in/around susceptibility genes/regions. This study explored whether the region of 4q22.1, which confers risk of developing osteoporosis in some populations, associated with bone mineral density and osteoporosis susceptibility in postmenopausal women of Han Chinese. We investigated 32 SNPs with minor allele frequencies ≥0.05 between 20 kb upstream and 20 kb downstream (40 kb window) of rs6532023, mapping in the 4q22.1 region, which was reported to be significantly associated with osteoporosis in previous studies. We found that rs6532023 was significantly associated with bone mineral density and osteoporosis (corrected p = 0.015) in our sample, including 440 cases and 640 controls, and allele G was supposed as a risk factor while T worked as a protective factor. Further genotype association analyses suggested a similar pattern (corrected p = 0.040). Additionally, analyses by haplotypes indicated that a haplotype block rs7683315-rs6532023-rs1471400-rs1471403 in the region associated with bone mineral density and osteoporosis (global p = 0.032), and risk haplotype A-G-G-C had almost 1.5-fold increased in the cases. To our knowledge, this is the first report to examine 4q22.1 region polymorphisms and osteoporosis in Han Chinese. Our results provide further evidence for an effect of the region of 4q22.1 on the etiology of osteoporosis and suggest that 4q22.1 may be a genetic risk factor for bone mineral density and osteoporosis.

## Introduction

Osteoporosis (OMIM166710) is a common skeletal disorder and can be severe, which is characterized by bone strength weak, and reduced bone mineral density (BMD) [1]. An imbalance in the regulation of bone remodeling causes the characteristic micro-architectural deterioration that compromises bone strength and leads to bone fragility increasing the fracture risk [2], [3]. As a major risk factor for osteoporosis, the measurement of BMD is used to define osteoporosis clinically [4]. In fact, previous studies proved that genetic factors play an important role in the pathogenesis of osteoporosis, and twin and family studies have emphasized the heritability of BMD [5]–[7]. Many genes have been associated with BMD [8]–[11]. Up to now, hot chromosomal regions focused on 5q31.1, 7q21.3, 11p12, 11q13.2, 12q13.11, 17q21.33, 20p12.3.

So far, a number of loci that contribute to osteoporosis and BMD regulation have been identified. Styrkarsdottir et al. [12] performed an association between 301,019 SNPs and bone mineral density of the hip or lumbar spine from 5,861 Icelandic subjects. The authors then tested for an association between 74 SNPs at 32 loci in replication sets of Icelandic, Danish, and Australian subjects. The authors then concluded that they discovered common sequence variants that are consistently associated with bone mineral density and with low-trauma fractures in 3 populations of European descent. Feng et al. [13] assessed the correlation between rs12218 polymorphism and osteoporosis in a population of Chinese women. Zhao et al. [14] indicate the relationship between osteoporosis and SNPs rs1038304, rs4870044, rs6929137, rs3130340, rs2306033, rs2273061 using different sample. Other researchers also explored the association between SNPs in different regions and BMD [15]–[17].

Estrada and others [18] identified 14 loci associated with bone mineral density and osteoporosis by Genome-wide meta-analysis, but there are only 4q22.1 and 11q13.2 with more functional evidence to support. Rs3736228 located in 11q13.2 site belongs to LRP5 gene, and LRP5 gene has been confirmed by more research as osteoporosis susceptibility genes [19]–[23]. Despite evidence of a strongly significant association within some populations, the genetic locus of 4q22.1 contributing to osteoporosis remains to be elucidated, and the underlying neurobiological mechanisms are largely unknown. Therefore, exploration of the possible association between the locus (4q22.1) and osteoporosis is necessary among other genetically independent populations

Researches also proved that osteoporosis showed the distinct age [24] and gender characteristics [25], [26], and it was more common in postmenopausal women. Up to date, the association between 4q22.1 and osteoporosis has not been investigated in Chinese Han population. In the current study we conducted the first genetic association study of the 4q22.1 region containing the SNP rs6532023, which is significantly associated with osteoporosis and BMD in the results of Estrada et al. [18], to determine whether or not the region is associated with osteoporosis in postmenopausal women of Chinese Han population based on a case-control study.

## Materials and Methods

### Subjects

The protocol of this study was approved by the Medical Ethics Committee of Xi'an Jiaotong University. All participants have completed written informed consent forms. All subjects used in this study were random unrelated Chinese Han individuals from Shaanxi Province, with no migration history within the previous three generations. Subjects with a history of bone disease, metabolic or endocrine disorders such as hyperthyroidism, hyperparathyroidism, diabetes mellitus, liver disease, renal disease, medications known to affect bone metabolism (e.g., corticosteroids, anticonvulsants, and heparin sodium) were excluded. Meanwhile, given that the possibility of osteoporosis resulted from obesity and the overall level of Asian body mass index (BMI), we ruled out subjects of BMI ≥27 in the study. None of the women had a history of taking medicines for the treatment of osteoporosis, such as active vitamin D3, bisphosphonates, SERM, or calcium. In total, 440 women (aged 49–78 years) with primary postmenopausal osteopenia or osteoporosis and 640 healthy age-matched women (aged 48–77 years) were recruited from the Second Affiliated Hospital of Xi'an Jiaotong University and Xi'an Honghui Hospital in this study.

### Measurement of bone mineral density and anthropometric baseline data

Dual-energy X-Ray absorptiometry (Lunar Expert 1313, Lunar Corp., USA) was used to assess BMD at the lumbar spine (L2–4) and femur neck. Bone mineral density was determined according to standard Lunar protocols. Bone mineral density was expressed in g/cm2 and as peak bone mass percentage in normal subjects (T-score) depending on the software used in the device. Results for the femur neck and lumbar spinal were classified into 3 groups according to World Health Organization criteria: normal (T score >−1.0 SD), osteopenia (T score −1.0 to −2.5 SD) and osteoporosis (T score <−2.5 SD). Subjects with osteopenia or osteoporosis (T score <−1.0 SD) were grouped into patients as both having low bone mass. In cases, three clinically distinct fracture definitions were used: (i) any type, consisting of low-trauma fractures at any skeletal site (except fingers, toes and skull) occurring after age 18 years, assessed by X-ray, radiographic report, clinical record, clinical interview and/or questionnaire, (ii) validated non-vertebral, consisting of fractures occurring after age 45 years, with diagnosis confirmed by hospital records and/or radiographs, and (iii) radiographic vertebral fractures, from lateral morphometry scored on X-rays. The first definition is most-inclusive, whereas the latter two are more stringent fracture definitions that are commonly used in randomized trials. Controls were defined as individuals without a history of any type of fracture.

The anthropometric baseline data of all subjects were obtained by measurement and questionnaire. All continuous variables were tested for skewness. The distribution of all variables was normal. The continuous parameters were presented as mean ± standard deviation, and comparisons of anthropometric characteristics of the subjects were performed using independent sample t-test for continuous variables (Table 1).

**Table 1:** Comparisons of anthropometric characteristics of the case and control groups.

Parameters	Assessment method	Case	Control	P-value
Age (years)	Questionnaire	62.6±6.9	62.1±7.6	0.898
Range of age (years)	Questionnaire	49–78	48–77	NA
Weight (kg)	Measured	62.5±6.6	62.9±7.2	0.772
Height (cm)	Measured	161.1±7.1	162.4±6.3	0.256
BMI (kg/m^2^)	Measured	24.1±3.5	23.9±3.7	0.734

Data are shown as mean ± SD, BMI body mass index.

### SNP selection and genotyping

**Figure 1:**
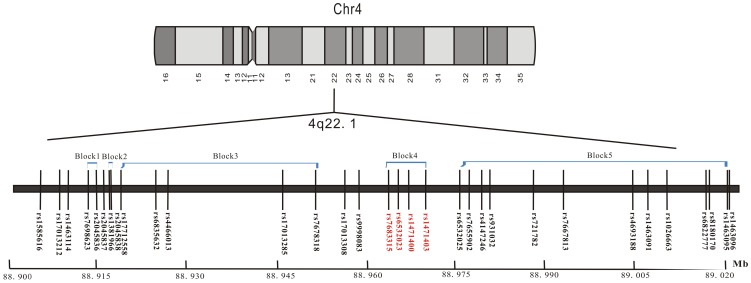
Distribution of 32 SNPs across the 4q22.1 region and their relationship with linkage disequilibrium blocks.

We searched for all SNPs with minor allele frequencies (MAF) >0.05 between 20 kb upstream and 20 kb downstream (40 kb window) of rs6532023 in the HapMap HCB database by Haploview [27]. We found 31 SNPs around rs6532023 (Figure 1), mapping in the 4q22.1 region, which was reported to be significantly associated with osteoporosis in a study conducted by Estrada et al. [18]. Therefore, we selected the 32 SNPs (Figure 1) for further analysis. Cases and controls were mixed on the same plates, and a double-blind procedure was used.

Peripheral blood was drawn from a vein into a sterile tube containing ethylenediamine tetraacetic acid (EDTA). Plasma samples were stored at −80°C. Genomic DNA was extracted from peripheral blood leukocytes according to the manufacturer's protocol (Genomic DNA kit, Axygen Scientific Inc., California, USA). DNA was stored at −20°C for SNP analysis. Genotyping was performed for all SNPs using the MassARRAY platform (Sequenom, San Diego, California, USA). Briefly, SNPs were genotyped using high-throughput, matrix-assisted laser desorption ionization–time-of-flight (MALDI–TOF) mass spectrometry. Next, the resulting spectra were processed using Typer Analyzer software (Sequenom), and genotype data were generated from the samples. As the final genotype call rate of each SNP was greater than 98% and the overall genotyping call rate was 98.8%, the reliability of further statistical analysis was ensured.

### Statistical analysis

Hardy–Weinberg equilibrium (HWE) for each SNP was assessed using GENEPOP v4.0 [28]. Allelic and genotypic association tests were performed using SPSS (Windows version release 15.0; SPSS Inc.; Chicago, IL, USA). Odds ratio (OR) and 95% confidence intervals (CIs) were also calculated. The D′ values for each pair of markers were calculated using the software program 2LD [29]. The haplotype frequencies were estimated using GENECOUNTING v2.2, which computes maximum-likelihood estimates of haplotype frequencies from unknown phase data by utilizing an expectation–maximization algorithm [30]–[33]. The significance of any haplotypic association with osteoporosis was then evaluated using a likelihood ratio test, followed by permutation testing that compared estimated haplotype frequencies in cases and controls [31], [33]. For the single marker association analysis, the p value was corrected in a Linkage Disequilibrium (LD) block considering the high conservation of Bonferroni correction (corrected p = p×n, the number of SNPs in a LD block). Differences were considered significant when the p value was less than 0.05. For haplotype analyses, the global p value was based on a comparison of the frequency distribution of all possible combinations of haplotypes indicated among cases and controls. Furthermore, we performed power calculations for case–control genetic association analyses using PGA v2.0 [34]. Our sample size can detect SNP and haplotype associations with 90% and 82% power, respectively, at a false positive rate of 5%.

## Results

32 SNPs in the 4q22.1 region were genotyped in 440 cases and 640 controls. The allele and genotype frequencies of all SNPs in cases and controls, including the results of the HWE test, are shown in Table 2 and Table S1. All SNPs were highly polymorphic in cases and controls, and the SNPs comply with Hardy-Weinberg equilibrium in both groups. We firstly conducted single SNP association analysis. When all of the samples were considered, we observed a significant association for rs6532023 (corrected p = 0.015). Genotype association analysis for rs6532023 suggested a similar pattern with a significant p value (corrected p = 0.040). As shown in Table 1, there was no statistical difference of the anthropometric baseline data between cases and controls. To investigate whether the associated SNP rs6532023 was affected by those factors or not, we performed adjustment for age, weight, height, BMI, spine BMD and femur BMD in each genotype group for the SNP rs6532023 in Table 3. We found significant difference of spine BMD and femur BMD among different rs6532023 genotype, suggesting that the association of rs6532023 persist after adjustment for those potential confounding factors.

**Table 2:** Allele and genotype frequency of single SNP association analysis.

Makers		Allele Freq. (%)		p-value^1^	Genotype Freq. (%)			p-value^1^	H-W E	OR^2^
SNP	ID/bp								p value	95%CI
SNP16	rs7683315	A	T		AA	AT	TT			
Case	88,773,095	66.8	33.2	0.365	42.7	48.2	9.1	0.546	0.069	1.089
Control		68.7	31.3		46.1	45.2	8.7		0.197	(0.906–1.308)
SNP17	rs6532023	G	T		GG	GT	TT			
Case	88,773,849	66.4	33.6	***0.004***	43.0	46.8	10.2	***0.010***	0.306	1.307
Control		60.2	39.8	***0.015***	34.6	51.2	14.2	***0.040***	0.083	(1.092–1.563)
SNP18	rs1471400	A	G		AA	AG	GG			
Case	88,774,247	61.2	38.8	0.462	36.2	50.0	13.8	0.632	0.268	1.073
Control		62.8	37.2		39.0	47.6	13.4		0.635	(0.899–1.280)
SNP19	rs1471403	C	T		CC	CT	TT			
Case	88,775,243	61.9	38.1	0.067	39.1	45.6	15.3	0.065	0.486	1.183
Control		65.8	34.2		42.1	47.4	10.5		0.179	(0.990–1.414)

CI: confidence interval; OR: odds ratio.

1. Significant p-values are in italic bold, and corrected p-values are underlined.

2. OR refers to risk allele odds ratio in cases and controls.

**Table 3:** Characteristics of the associated SNP rs6532023 in the total group of subjects.

SNP	rs6532023			P
Genotype	GG	GT	TT	value
Number	410	534	136	NA
Age (years)	62.8	62.5	61.2	0.963
Weight (kg)	62.9	62.4	63.6	0.315
Height (cm)	162.4	161.4	162.1	0.195
BMI (kg/m^2^)	23.9	24.0	24.2	0.717
Spine BMD (g/cm^2^)	0.838	0.932	0.884	<0.001
Femur BMD (g/cm^2^)	0.811	0.865	0.823	<0.001

Data are shown as mean ± SD, BMI body mass index, BMD bone mineral density.

Because rs7683315, rs1471400 and rs1471403 were in the same Linkage Disequilibrium (LD) block containing rs6532023 from analysis of HapMap dataset, we selected the four SNPs (rs7683315, rs6532023, rs1471400 and rs1471403) for further analysis. Taking into account that rs6532023 in the region of 4q22.1 yielded a significant association, we performed linkage disequilibrium (LD) estimation for the 4 SNPs (rs7683315, rs6532023, rs1471400, and rs1471403) in the region of 4q22.1. Table 4 presents the results of LD tests (noted as D′ and r2) between pairs of these 4 SNPs for the respective control groups. According to these results (D′>0.8), LD was observed in the 4-SNP linkage disequilibrium estimation. When combining the allele frequency data with the LD, the associated rs6532023 was observed between patients and controls for another 3 SNPs (rs7683315, rs1471400, and rs1471403) in the same LD region. Therefore, we next carried out haplotypic association analyses of rs7683315-rs6532023-rs1471400-rs1471403, as shown in Table 5. Tests of the 4-marker haplotype analysis provided evidence of a significant association with osteoporosis (p = 0.032, global permutation). Some haplotypes showed a significant association with osteoporosis in female. In detail, HAP3 was significantly associated with osteoporosis and the frequency was increased almost 1.5-fold (p = 0.009) in cases. Due to the higher frequencies in controls, a protective effect may be implicated by HAP4 (Table 5).

**Table 4:** Estimation of LD between each pair of loci.

	rs7683315	rs6532023	rs1471400	rs1471403
rs7683315	-	0.526	0.617	0.737
rs6532023	0.874	-	0.780	0.518
rs1471400	0.896	0.933	-	0.635
rs14714003	0.917	0.812	0.851	-

D′-value are shown below the subtraction sign, and r2-value are shown above the subtraction sign.

**Table 5:** Haplotypes frequency and association analysis.

Haplotype					Genecounting (frequency %)			
ID	SNP1	SNP2	SNP3	SNP4	Case	Control	p-value^1^	Global p^2^
HAP1	A	G	A	C	50.2	51.3	0.615	***0.032***
HAP2	T	T	G	T	25.9	24.4	0.419	
***HAP3*** 3	A	G	G	C	9.41	6.38	***0.009***	
***HAP4*** 3	T	T	A	T	2.94	4.68	***0.043***	
HAP5	A	G	A	T	2.15	2.01	0.838	

Significant p-values are in italic bold. Haplotypes are not shown, if frequency less than 2%.

1. Based on 10000 permutations.

2. Based on comparison of frequency distribution of all haplotypes for the combination of SNPs.

3. Haplotypes in italic bold are the significant ones in the study.

## Discussion

The purpose of the present study was to investigate the relationship between the region of 4q22.1 containing rs6532023 and osteoporosis susceptibility. With the fast development and extensive use of Genome-wide association study, more and more osteoporosis susceptibility loci will be reported. However, their observations will need further evidence from other independent populations. In this study, we present evidence of association between rs6532023 within 4q22.1 and osteoporosis, where some significantly associated haplotypes also appeared. These lend additional support to the positive association results for osteoporosis and 4q22.1. Several lines of evidence suggest that the observed association is unlikely to be an artifact. First, both the single SNP and the haplotype-based association analyses support the association. Second, population stratification is the unlikely explanation because all of our samples are from the same geographical region. Finally, similar results were obtained from the studies of Estrada et al.'s and ours, reaffirming the observed association.

To examine if the common risk variants exist in genetically independent populations, we compared our results with those of Estrada et al. [18]. In both studies, rs6532023 showed significant association with osteoporosis and BMD, and G allele of rs6532023 is a risk allele. In addition, a risk haplotype HAP3 that included rs6532023 showed significant positive association with osteoporosis in our studies. Collectively, the consistency between these two studies of different populations provides strong evidence that the polymorphism of the region of 4q22.1 may be involved in osteoporosis susceptibility. However, we also observed some differences between two studies. The odds ratios of rs6532023 was 1.307 in our data compared with 1.06 in the report by Estrada et al. [18], and the risk allele frequency in cases was lower in Han Chinese (0.664 in our samples, 0.67 in the Estrada et al.'s samples). Differences in results between our study and Estrada et al.'s study may be caused by ethnic differences and female samples in our study, even though there are some similarities in the general association pattern of both studies.

The ability to draw conclusions regarding associations based on the analysis of individual SNPs is limited [35]. Therefore, to obtain stronger statistical evidence for the association we performed haplotype analysis. Haplotype analysis uses additional information on linkage between markers typed. The results of haplotype frequency estimation for 4-SNP (rs7683315-rs6532023-rs1471400-rs1471403) showed significant associations with osteoporosis (p = 0.032, global permutation). We noticed that two significant haplotypes have relatively low frequencies, which may increase the inaccuracy of haplotype frequency estimation and lead to false-positive inference.

4q22.1 implicates the region containing multiple genes, where the SNP (rs6532023) was found to be strongly significant association with osteoporosis and BMD in some populations. Actually, the 4q21.1 region contains a cluster of structurally and phylogenetically related genes encoding matricellular phosphoglycoproteins with function in bone formation and growth [36]. The associated rs6532023 is located 6 Kb to the matrix extracellular phosphoglycoprotein (MEPE) gene (also known as osteoblast/osteocyte factor 45), 41 Kb to the integrin-binding sialoprotein (IBSP) gene and 123 Kb to the secreted phosphoprotein 1 (SPP1) gene, also known as osteopontin. IBSP and SPP1 are highly expressed in osteoblasts, osteoclasts and hypertrophic chondrocytes. MEPE is predominantly expressed by osteocytes in human bone, playing an inhibitory role in bone formation. All three genes display diverse skeletal phenotypes in mice knock out (KO) models. MEPE (Of 45) KO show increased bone mass and inhibition of age-related bone loss [37], IBSP KO show high trabecular bone density with low bone turnover but respond to bone loss caused by disuse [5] and the SPP1 KO have high trabecular bone mass and is resistant to bone loss [38]. As a result, the same alleles of the same SNP in the region of 4q22.1 as reported by Estrada et al. [18] significantly replicated in our sample sets. However, all our interesting findings should therefore be considered preliminary, and additional follow-up studies are required including high density mapping and deep sequencing in other genetically independent populations to find possible causal variants.

A major limitation of the current study is that the sample size of our population, especially the case number, seems to be modest. Additionally, it is important to note that we have not attempted to genotype a larger set of SNPs in the region; rather we have selected a subset of 31 SNPs neighboring rs6532023 reported by Estrada et al. [18]. The justification for the sample size and the assessed SNPs subset might be weak, but it has got sufficient statistical power to detect SNP and Haplotype association in power analysis and been scientifically reasonable for a replication association study in different populations. Therefore, these data should be interpreted with caution, since they need to be confirmed in a larger sample in order to properly detect whether possible associations exit or not, especially in man.

In conclusion, our work provides supportive evidence for the association of 4q22.1 with osteoporosis susceptibility. Moreover, we have also confirmed the previous reports suggesting that the region of 4q22.1 is likely responsible for osteoporosis and may play an important role in the etiology of osteoporosis and BMD regulation. Given the complex patterns of association findings in complex disorders such as sex specificity and genetic heterogeneity, further inquiries and wider replications are still needed in men, especially in studies using different ethnic samples.

## Supporting Information

Table S1 **Allele and genotype frequency of all SNPs association analyses.**
(DOC)
