# Supplementary material for: 4q22.1 Contributes to Bone Mineral Density and Osteoporosis Susceptibility in Postmenopausal Women of Chinese Han Population
Source: PLoS One. 2013 Nov 21;8(11):e80165. doi: 10.1371/journal.pone.0080165 (PMC3836996; doi:10.1371/journal.pone.0080165)
Supplement: Table S1 — Allele and genotype frequency of all SNPs association analyses. (DOC) [file pone.0080165.s001.doc]

Table S1 Allele and genotype frequency of all SNPs association analyses

| Makers  SNP ID / bp | | Allele Freq. (%) | | p-value | Genotype Freq. (%) | | | p-value | H-W E  p value | OR^1^  95%CI |
| --- | --- | --- | --- | --- | --- | --- | --- | --- | --- | --- |
| SNP1 | rs1585616  88,753,045 | C | T |  | CC | CT | TT |  |  |  |
| Case |  | 21.3 | 78.7 | 0.368 | 3.7 | 35.2 | 61.1 | 0.157 | 0.295 | 1.103  (0.896-1.357) |
| Control |  | 22.9 | 77.1 |  | 6.2 | 33.4 | 60.4 |  | 0.171 |  |
| SNP2 | rs17013212  88,754,060 | C | T |  | CC | CT | TT |  |  |  |
| Case |  | 69.1 | 30.9 | 0.299 | 48.0 | 42.2 | 9.8 | 0.429 | 0.805 | 1.104  (0.916-1.332) |
| Control |  | 71.2 | 28.8 |  | 51.9 | 38.6 | 9.5 |  | 0.137 |  |
| SNP3 | rs1463114  88,754,681 | C | T |  | CC | CT | TT |  |  |  |
| Case |  | 84.5 | 15.5 | 0.624 | 72.2 | 24.6 | 3.2 | 0.888 | 0.202 | 1.062 (0.836-1.349) |
| Control |  | 85.3 | 14.7 |  | 73.4 | 23.8 | 2.8 |  | 0.197 |  |
| SNP4 | rs7698623  88,755,828 | C | T |  | CC | CT | TT |  |  |  |
| Case |  | 76.2 | 23.8 | 0.093 | 57.3 | 37.8 | 4.9 | 0.120 | 0.377 | 1.197 (0.975-1.470) |
| Control |  | 79.3 | 20.7 |  | 63.4 | 31.8 | 4.8 |  | 0.427 |  |
| SNP5 | rs2045836  88,756,245 | C | T |  | CC | CT | TT |  |  |  |
| Case |  | 12.4 | 87.6 | 0.444 | 1.9 | 21.0 | 77.1 | 0.719 | 0.484 | 1.114 (0.861-1.441) |
| Control |  | 13.5 | 86.5 |  | 2.2 | 22.6 | 75.2 |  | 0.413 |  |
| SNP6 | rs2045837  88,756,675 | C | T |  | CC | CT | TT |  |  |  |
| Case |  | 83.3 | 16.7 | 0.630 | 69.8 | 27.0 | 3.2 | 0.888 | 0.535 | 1.058  (0.842-1.329) |
| Control |  | 82.5 | 17.5 |  | 68.6 | 27.8 | 3.6 |  | 0.346 |  |
| SNP7 | rs1381966  88,756,978 | C | T |  | CC | CT | TT |  |  |  |
| Case |  | 34.8 | 65.2 | 0.849 | 10.9 | 47.8 | 41.3 | 0.788 | 0.263 | 1.018 (0.850-1.219) |
| Control |  | 34.4 | 65.6 |  | 11.6 | 45.6 | 42.8 |  | 0.793 |  |
| SNP8 | rs2045838  88,757,008 | A | G |  | AA | AG | GG |  |  |  |
| Case |  | 71.6 | 28.4 | 0.655 | 49.7 | 43.8 | 6.5 | 0.743 | 0.106 | 1.042 (0.862-1.259) |
| Control |  | 70.7 | 29.3 |  | 49.2 | 43.0 | 7.8 |  | 0.338 |  |
| SNP9 | rs17712558  88,757,703 | G | T |  | GG | GT | TT |  |  |  |
| Case |  | 33.2 | 66.8 | 0.491 | 9.3 | 47.8 | 42.9 | 0.255 | 0.103 | 1.066 (0.889-1.278) |
| Control |  | 34.6 | 65.4 |  | 12.3 | 44.6 | 43.1 |  | 0.714 |  |
| SNP10 | rs6835632  88,759,690 | C | T |  | CC | CT | TT |  |  |  |
| Case |  | 65.1 | 34.9 | 0.363 | 40.6 | 49.0 | 10.4 | 0.489 | 0.100 | 1.085  (0.907-1.298) |
| Control |  | 63.2 | 36.8 |  | 39.2 | 48.0 | 12.8 |  | 0.419 |  |
| SNP11 | rs4466013  88,760,413 | A | G |  | AA | AG | GG |  |  |  |
| Case |  | 83.5 | 16.5 | 0.595 | 70.5 | 26.0 | 3.5 | 0.895 | 0.237 | 1.059  (0.839-1.338) |
| Control |  | 84.4 | 15.6 |  | 71.9 | 25.0 | 3.1 |  | 0.200 |  |
| SNP12 | rs17013285  88,767,008 | A | G |  | AA | AG | GG |  |  |  |
| Case |  | 25.8 | 74.2 | 0.405 | 7.9 | 35.8 | 56.3 | 0.317 | 0.173 | 1.091 (0.895-1.329) |
| Control |  | 24.2 | 75.8 |  | 5.6 | 37.2 | 57.2 |  | 0.724 |  |
| SNP13 | rs7678318  88,768,888 | C | T |  | CC | CT | TT |  |  |  |
| Case |  | 25.5 | 74.5 | 0.620 | 5.2 | 40.6 | 54.2 | 0.830 | 0.150 | 1.045  (0.859-1.271) |
| Control |  | 26.4 | 73.6 |  | 6.1 | 40.6 | 53.3 |  | 0.258 |  |
| SNP14 | rs17013308  88,770,559 | G | T |  | GG | GT | TT |  |  |  |
| Case |  | 76.2 | 23.8 | 0.525 | 57.7 | 37.0 | 5.3 | 0.791 | 0.673 | 1.071  (0.874-1.312) |
| Control |  | 77.4 | 22.6 |  | 59.4 | 36.0 | 4.6 |  | 0.463 |  |
| SNP15 | rs9998083  88,771,391 | A | C |  | AA | AC | CC |  |  |  |
| Case |  | 81.2 | 18.8 | 0.223 | 66.5 | 29.4 | 4.1 | 0.488 | 0.437 | 1.150  (0.919-1.438) |
| Control |  | 83.3 | 16.7 |  | 69.9 | 26.8 | 3.3 |  | 0.353 |  |
| SNP20 | rs6532025  88,777,194 | A | G |  | AA | AG | GG |  |  |  |
| Case |  | 25.8 | 74.2 | 0.560 | 5.9 | 39.8 | 54.3 | 0.817 | 0.407 | 1.060  (0.871-1.292) |
| Control |  | 24.7 | 75.3 |  | 5.2 | 39.0 | 55.8 |  | 0.220 |  |
| SNP21 | rs7655902  88,777,743 | C | T |  | CC | CT | TT |  |  |  |
| Case |  | 38.8 | 61.2 | 0.488 | 14.9 | 47.8 | 37.3 | 0.810 | 0.892 | 1.059 (0.888-1.262) |
| Control |  | 40.2 | 59.8 |  | 16.2 | 48.0 | 35.8 |  | 0.967 |  |
| SNP22 | rs4147246  88,778,249 | C | G |  | CC | CG | GG |  |  |  |
| Case |  | 39.8 | 60.2 | 0.910 | 16.7 | 46.2 | 37.1 | 0.699 | 0.452 | 1.012  (0.849-1.207) |
| Control |  | 39.5 | 60.5 |  | 15.2 | 48.6 | 36.2 |  | 0.670 |  |
| SNP23 | rs931032  88,778,446 | C | T |  | CC | CT | TT |  |  |  |
| Case |  | 76.2 | 23.8 | 0.199 | 57.3 | 37.8 | 4.9 | 0.367 | 0.377 | 1.151 (0.938-1.412) |
| Control |  | 78.6 | 21.4 |  | 61.6 | 34.0 | 4.4 |  | 0.787 |  |
| SNP24 | rs721782  88,781,437 | G | T |  | GG | GT | TT |  |  |  |
| Case |  | 74.9 | 25.1 | 0.666 | 55.6 | 38.6 | 5.8 | 0.935 | 0.577 | 1.037 (0.852-1.263) |
| Control |  | 74.1 | 25.9 |  | 54.5 | 39.2 | 6.3 |  | 0.591 |  |
| SNP25 | rs7667813  88,783,177 | C | T |  | CC | CT | TT |  |  |  |
| Case |  | 9.6 | 90.4 | 0.278 | 1.3 | 16.6 | 82.1 | 0.312 | 0.360 | 1.196 (0.887-1.615) |
| Control |  | 8.2 | 91.8 |  | 0.8 | 14.8 | 84.4 |  | 0.668 |  |
| SNP26 | rs4693188  88,787,174 | A | G |  | AA | AG | GG |  |  |  |
| Case |  | 37.6 | 62.4 | 0.495 | 13.3 | 48.6 | 38.1 | 0.703 | 0.454 | 1.064  (0.891-1.271) |
| Control |  | 36.2 | 63.8 |  | 11.7 | 49.0 | 39.3 |  | 0.124 |  |
| SNP27 | rs1463091  88,788,036 | C | T |  | CC | CT | TT |  |  |  |
| Case |  | 32.4 | 67.6 | 0.664 | 11.0 | 42.8 | 46.2 | 0.899 | 0.630 | 1.043 (0.869-1.253) |
| Control |  | 33.3 | 66.7 |  | 11.7 | 43.2 | 45.1 |  | 0.486 |  |
| SNP28 | rs1026663  88,789,140 | C | T |  | CC | CT | TT |  |  |  |
| Case |  | 56.3 | 43.7 | 0.312 | 30.9 | 50.8 | 18.3 | 0.586 | 0.497 | 1.094 (0.920-1.301) |
| Control |  | 58.4 | 41.6 |  | 33.4 | 50.0 | 16.6 |  | 0.462 |  |
| SNP29 | rs6822777  88,791,395 | A | G |  | AA | AG | GG |  |  |  |
| Case |  | 81.7 | 18.3 | 0.393 | 67.3 | 28.8 | 3.9 | 0.671 | 0.439 | 1.107 (0.884-1.387) |
| Control |  | 83.1 | 16.9 |  | 69.4 | 27.4 | 3.2 |  | 0.536 |  |
| SNP30 | rs8180170  88,791,579 | C | T |  | CC | CT | TT |  |  |  |
| Case |  | 82.3 | 17.7 | 0.855 | 68.4 | 27.8 | 3.8 | 0.933 | 0.337 | 1.021 (0.815-1.279) |
| Control |  | 82.6 | 17.4 |  | 68.6 | 28.0 | 3.4 |  | 0.512 |  |
| SNP31 | rs1463095  88,792,621 | A | G |  | AA | AG | GG |  |  |  |
| Case |  | 30.6 | 69.4 | 0.500 | 9.3 | 42.6 | 48.1 | 0.744 | 0.950 | 1.071 (0.888-1.291) |
| Control |  | 29.2 | 70.8 |  | 8.2 | 42.0 | 49.8 |  | 0.690 |  |
| SNP32 | rs1463096  88,792,715 | C | G |  | CC | CG | GG |  |  |  |
| Case |  | 43.8 | 56.2 | 0.451 | 20.5 | 46.6 | 32.9 | 0.627 | 0.262 | 1.068  (0.899-1.270) |
| Control |  | 45.4 | 54.6 |  | 21.0 | 48.8 | 30.2 |  | 0.692 |  |

CI: confidence interval; OR: odds ratio

1. OR refers to risk allele odds ratio in cases and controls.
